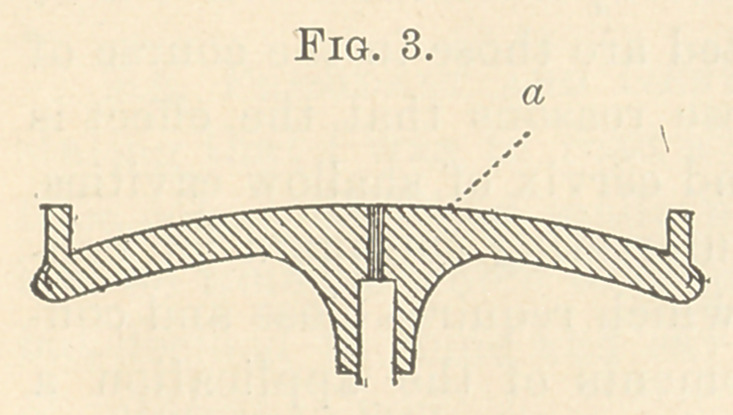# Electrical Osmosis in the Treatment of Acute Dentinal Sensibility

**Published:** 1896-06

**Authors:** Louis Jack


International Dental Journal.

Vol. XVII. June, 1896. No. 6.

### Original Communications.[1]

### ELECTRICAL OSMOSIS IN THE TREATMENT OF ACUTE DENTINAL SENSIBILITY.

BY LOUIS JACK, D.D.S.

In the application of electrical osmosis for obtunding dentinal sensibility I have had the experience of a variety of cases in the past three weeks.

The effect of cocaine so administered is marvellous. In every instance of the most extreme sensibility where I have used it in this manner the result has been profound. In most cases the obtundation was absolute,—in some after the caries had been removed and considerable progress made towards the retentive formation, a normal degree of sensation appeared.

The apparatus I have used is the No. 11 of the Chloride of Silver Dry Cell Battery Company. This form contains in one case any desired number of cells up to fifty, a milammeter with three scales, a cell selector, and a controller, with the usual cut-offs, and the ordinary medical electrodes.

Fig. 1 shows this apparatus as adapted to medical purposes. For dental use the drawer is not required. The reduction of the height of the case by excluding the drawer makes it more convenient and in better proportion.

I was induced to select this apparatus for the reason that it

[1] The editor and publishers are not responsible for the views of authors of papers published in this department, nor for any claim to novelty, or otherwise, that may be made by them. No papers will be received for this department that have appeared in any other journal published in the country.

is well known that the chloride of silver cell is the kind best adapted to medical purposes as having an agreeable ratio between the voltage and the ampèrage, the voltage of each cell being one and the ampèrage between one-fifth and one-fourth. It is always constant, which means that there is no polarization, and that it does not

Fig. 1.

decline in power until nearly exhausted. They emit no fumes and there is no "creeping" of the electrolyte to interfere with the action. Moreover the cells are "dry."

The controller is the most important adjunct of this apparatus. It is stated that its highest resistance is ninety thousand ohms. On its surface are one hundred and twelve contact pins which may be put in the circuit. From the first to the last the gradation of the reducing resistance as the switch is advanced is extremely regular. This permits the current in ampèrage to be slowly increased as the indications permit. The initial voltage is determined by the number of cells selected. Some cases requiring only ten or fifteen cells, others twenty. In only two instances have I used twenty-five. The difference in the number of cells required appears to depend upon the varying resistance of the tooth being operated upon. Ten cells are sufficient for children, and for teeth apparently soft. More than fifteen cells become necessary for adults where the dentine is dense.

Storage cells, as stated by Dr. Brown in the April number of the INTERNATIONAL DENTAL JOURNAL, used with the Willms controller,—which is the same as the one above mentioned,—would be equally constant, but the ratio of ampèrage to the voltage of each cell should be nearly the same as above indicated. This would be nearly satisfied by type B of the "chloride accumulator" having three plates three inches by three inches to each cell, normal charge rate five-eighths of an ampère. Discharge in ampères for eight hours, five-eighths. This would equal six hundred milliampère hours. As the rate of discharge during electrical osmosis is not usually greater than three milliampères of current, this should theoretically permit eight hundred applications of fifteen minutes each. Of storage cells twelve would be required. The only question concerning this source of force is the embarrassment connected with charging.

As to the application of cocaine and of the electrodes, I follow the methods stated by Dr. Gillette in his articles.

FIG. 2.

Fig. 2 represents the anodal electrode with its platinum point. This instrument is jointed at *a* by screw connections to enable the handles to receive various sizes and shapes of points. The battery cords fit the socket at the distal end.

The cathodal electrode is represented in section by Fig. 3. The surface *a* is a plate covered with platinum-foil to prevent oxidation. The connection is made with the cords in the usual manner on the reverse side. This form is adapted to be held in contact with the face by the projection on the reverse side passing through an opening in the band which supports the rubber dam. It can also be held by the finger.

FIG. 3.

In use the recess is filled with a disk of amadou (spunk) or of thick blotting paper, which is first wetted with a solution of sodium chloride. The usual sponge electrodes soon become disagreeable, and, further, it is more cleanly to use a fresh disk for each person.

As to the placement of the cathodal electrode it has appeared better to put it on the cheek for the upper teeth and on the neck for the lower; in each case it is placed near the ear. I have not observed reddening of the skin which some have noticed. Should the person have much adipose tissue on the face which much increases the resistance, it is better to have this electrode held in the hand.

When the electrodes are placed the switch of the controller is very slowly advanced until the movement of the eyelid indicates slight, irritation of the tooth. This is to be distinguished from the first impact of the current, as even at ten cells the first contact pin is felt notwithstanding the high resistance of the controller, but after this is passed the switch may be carried on the circle nearly forty-five degrees before indication of irritation appears. As the sensation passes off, the switch is slowly advanced a pin at a time and so continued until by a more rapid lessening of the resistance no irritation appears. Usually at this point in the administration nearly all the resistance may be taken off. If this can be done it is conclusive that relief has been attained. The switch is then carried back to zero when the electrodes are removed.

Since I have been using this means it has not failed in a single instance to overcome the sensibility so as to permit the complete removal of the caries and the preparation of the cavity. In some instances normal sensibility of healthy dentine is observed in the preparation of the margins during the formation of the retaining grooves. When this is the case it is usually found at the margin towards the occlusal aspect. When we reflect that as the remedy is carried on a line somewhat coincident with the direction of the current, and as the course of the current is from the broad surface of the cavity towards the apex of the tooth it is to be expected that the parts most affected are those in the course of the current. It is apparently for these reasons that the effect is the most pronounced on the bottom and cervix of shallow cavities.

There is another important and interesting consideration connected with the effect of the current which requires close and continued observation. After a few moments of the application a sensation is produced in the tooth which is often compared by the patient with the effect of subjecting the tooth to changes of temperature. When it is considered that the resistance of the teeth, on account of their density and composition, is high, as is shown by the great disparity between the amperage of the current tested when at short circuit and as shown by the milammeter when the tooth is included in the circuit, it is indicated that this sensation is brought about by some elevation of the temperature of the tooth, caused by its electrical resistance. This rise of temperature is probably modified by the evaporation taking place from the aqueous solution in the cavity.

The period required to produce the effect has usually been about twelve minutes, in some instances in clinical experiments, when the conditions were extreme, the continuance has been greater.

As to the cocaine solution I prefer sixteen to twenty-four per cent. The cocaine hydrochloride pellets of Wyeth Brothers, which are everywhere procurable, contain one and one-fifth grains. One of these to five minims of water gives twenty-four per cent., to seven and one-half minims sixteen per cent., to ten minims twelve per cent. The graduated minim measures are a most convenient vessel to make the solution in.

After repeated trials I have come to consider cocaine citrate as superior to the hydrochloride; the former being efficient in dense tissue when the effect of the latter has not been complete. The citrate is at present procurable of Merck & Co., of New York.

NOTE.—For the recent literature of this subject, see "The International System of Electro-Therapeutics," Section C, pp. 1 to 20. *Dental Cosmos*, June 1895, and March 1896, p. 210, *et seq.* INTERNATIONAL DENTAL JOURNAL, February and April, 1896. "Proceedings of the American Dental Association for 1895."

## Figures and Tables

**Fig. 1. f1:**
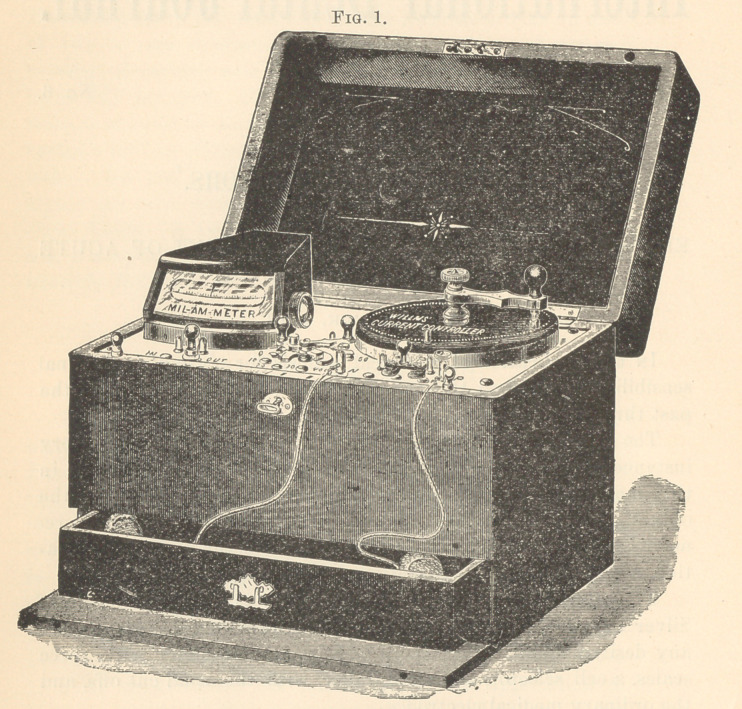


**Fig. 2. f2:**
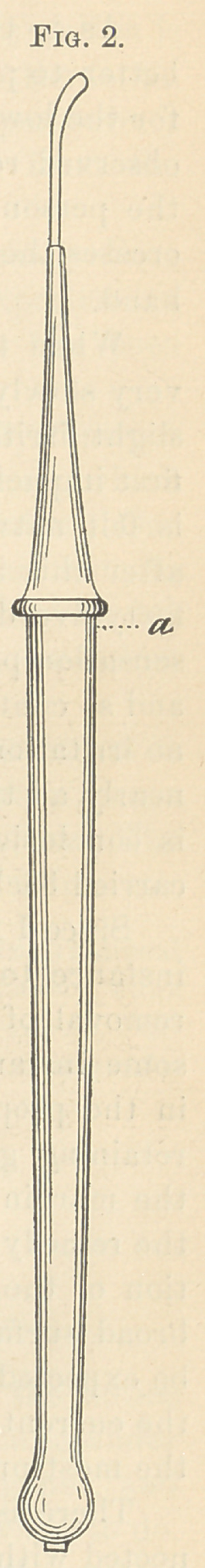


**Fig. 3. f3:**